# Ultra-Miniaturized Dual-Band MIMO Antenna for Biomedical Implantable Devices in Wireless Health Monitoring Systems

**DOI:** 10.3390/bios16030163

**Published:** 2026-03-14

**Authors:** Tahir Bashir, Shunbiao Chen, Guanjie Feng, Yunqi Cao, Wei Li

**Affiliations:** 1 College of Integrated Circuit Science and Engineering, Nanjing University of Posts and Telecommunications, Nanjing 210023, China; tahir.bashir@njupt.edu.cn (T.B.); b22022219@njupt.edu.cn (S.C.); b22030509@njupt.edu.cn (G.F.); 2 College of Control Science and Engineering, Zhejiang University, Hangzhou 310027, China; caoyunqi@zju.edu.cn; 3 School of Mechanical Science and Engineering, Huazhong University of Science and Technology, Wuhan 430074, China

**Keywords:** implantable antenna, MIMO, capsule endoscopy, pacemaker

## Abstract

This paper proposed an ultra-miniaturized four-port dual-band multi-input multi-output (MIMO) antenna designed for wireless biomedical implantable devices, including wireless capsule endoscopy (WCE) and cardiac leadless pacemakers. The antenna supports operation in the wireless medical telemetry service (WMTS) band of 1.395–1.4 GHz and the industrial, scientific, and medical (ISM) band of 2.4–2.4835 GHz for wireless power transfer and data telemetry applications. Miniaturization is achieved through a partial meandered structural configuration, yielding an overall size of 8 × 6.4 × 0.5 mm^3^. The antenna is encapsulated within implantable biomedical devices containing batteries, sensors, and electronic components, and evaluated in both homogeneous and realistic heterogeneous body phantoms, including the large intestine and heart. The full-wave electromagnetic simulation results demonstrate good performance, including reflection coefficients of −31.19 dB and −30.07 dB, gains of −27.5 dBi and −17.5 dBi, −10 dB impedance bandwidths of 170 MHz and 370 MHz, mutual coupling below 20 dB, and fractional bandwidths of 12.2% and 15.1% at 1.4 GHz and 2.45 GHz, respectively. Specific absorption rate (SAR) analysis satisfies implantation safety limits. Link budget analysis confirms reliable communication over distances more than 20 m in both frequency bands with high-data rates up to 100 Mbps. MIMO channel parameters such as envelope correlation coefficient (ECC), diversity gain (DG), channel capacity loss (CCL), and total active reflection coefficient (TARC) confirm the usefulness of the proposed MIMO antenna. Consequently, the proposed MIMO antenna emerges as a highly promising candidate with, ultra-miniaturization, isolation, multiband operation ability with omnidirectional-like radiation pattern characteristics for several biomedical implants in wireless health monitoring systems.

## 1. Introduction

**Figure 1:**
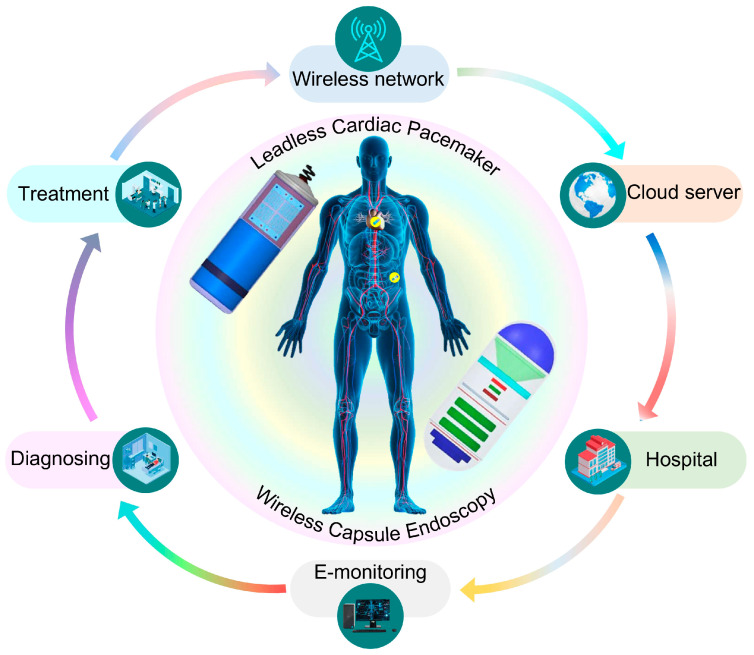
An overview of the proposed miniaturized dual-band MIMO antenna designed for integration into bio-telemetric systems.

Recent advancements in medical electronic technologies have gained significant re-search interest in biomedical implantable devices (IMDs). These devices support essential biomedical functions, such as physiological monitoring, wireless data transmission, and neural stimulation, thereby enabling early diagnosis and improved treatment outcomes [1]. IMDs consist of several key components, including a container, batteries, sensors, circuitry, and implantable antennas. Among these elements, the implantable antenna plays a significant role in ensuring reliable communication between internal implantable and external receiver antennas [2]. However, their design presents several challenges, including antenna miniaturization, effective impedance matching, reliable high-speed data transmission, omnidirectional-like radiation performance, and adherence to human safety standards. With the growing demand for compact and advanced IMBDs, there is a critical need for miniaturized, multiband antennas capable of addressing the challenges caused by limited device dimension and diverse frequency requirements in biomedical applications. Antenna miniaturization is essential due to the spatial constraints within both IMBDs and the human body. Furthermore, implantable antennas must be designed to operate efficiently in the highly lossy biological environment, while avoiding discomfort to the patient and preventing interference with internal IMBD components. Simultaneously, modern IMBDs are increasingly required to operate across multiple frequency bands to support a variety of communication functions, including wireless power transfer, data transmission, and remote control [3], see Figure 1.

To address these challenges, prior research has investigated single-input single-output (SISO) implantable antennas [4,5,6,7]. Nevertheless, SISO systems inherently exhibit limited spectral efficiency and reduced data throughput. Consequently, MIMO implantable antennas have emerged as a more robust solution [8,9,10,11]. Despite the incorporation of multiple radiating elements, many existing MIMO antennas are restricted to single-band operation and occupy relatively large volumes, preventing them from fully satisfying the multi-frequency and miniaturization requirements of advanced IMBDs. Extensive efforts have been dedicated to attaining multiband capabilities, high data rates, and compactness in implantable antenna systems [12,13,14,15,16,17,18,19,20]. For further miniaturization, conformal antenna designs employing the inner and outer surfaces of capsule-type devices have also been examined [21,22,23]. While these structures contribute to reduced antenna dimensions, their comparatively large overall size confines them to capsule-based platforms, limiting applicability to broader IMBD systems with critical space constraints. Existing implantable antenna designs reported in the literature face one or more challenges, such as large physical size, frequency operation, insufficient isolation, directional radiation characteristics, limited bandwidth, and compatibility limited to specific devices or applications. Thus, there remains a significant need for an implantable antenna offering miniaturization, multiband operation, good isolation, adequate bandwidth, and adaptability across several IMBDs.

This study proposes a highly miniaturized four-port dual-band implantable MIMO antenna designed for capsule endoscopy and cardiac leadless pacemaker systems. Designing implantable antennas requires a balance between operating frequency and performance. Lower-frequency bands, such as MICS and MedRadio, provide better tissue penetration and lower power consumption, while higher-frequency bands, including WMTS and ISM bands, support compact antenna design, wider bandwidth, and higher data transmission rates. To achieve these properties and support multiple communication protocols, the proposed MIMO antenna operates in both the WMTS 1.4 GHz band and the ISM 2.45 GHz band, enabling wireless power transfer and data telemetry applications. The proposed antenna features an ultra-compact structural configuration, including a partial-meandered hybrid patch geometry, multiband operation using a limited number of identical patch slots, and a fully connected ground plane. The proposed four-element MIMO antenna occupies a minimal volume of 25.6 mm^3^ and achieves good isolation between elements without the use of external components. The four-element MIMO configuration achieves good performance, including reflection coefficient, good inter-element isolation, gain, −10 dB impedance bandwidth, fractional bandwidth, and omnidirectional-like radiation characteristics. To validate applicability across various biomedical platforms, the antenna was encapsulated within IMBDs and evaluated in both homogeneous and heterogeneous tissue models. Safety compliance was verified through SAR analysis to ensure adherence to human body exposure limits. Additionally, a link margin analysis was conducted to analyze the reliability of wireless communication, confirming data transmission over several meters at high data rates (100 MBps). Key MIMO channel parameters including ECC, DG, CCL, and TARC were further analyzed, validating the antenna’s suitability for biomedical implantation. Overall, the proposed structure demonstrates compactness, structural efficiency, a multi-functional antenna system and reliable performance under implantable environment constraints. Table 1 compares the proposed MIMO antenna with existing implantable MIMO designs, emphasizing its advantages for biomedical applications. This study is based exclusively on numerical modeling and full-wave electromagnetic simulations in both homogeneous and heterogeneous body phantom models. The proposed antenna has been evaluated through comprehensive full-wave electromagnetic simulations using both homogeneous and heterogeneous human tissue models to represent realistic implantation environments. In addition, SAR analysis, link budget evaluation, and MIMO channel parameters have been investigated to assess safety and communication reliability under biomedical conditions. It is worth noting that several studies in the literature have also reported implantable antenna designs validated primarily through numerical and full-wave simulation analysis during the design and feasibility stage [24,25,26,27,28]. Although experimental validation using tissue-equivalent phantoms would provide further verification, such measurements for ultra-miniaturized multi-port implantable antenna configurations require specialized fabrication and controlled measurement facilities and will therefore be considered in future work.

## 2. Capsule-like Implantable Devices and Simulation Setup

**Figure 2:**
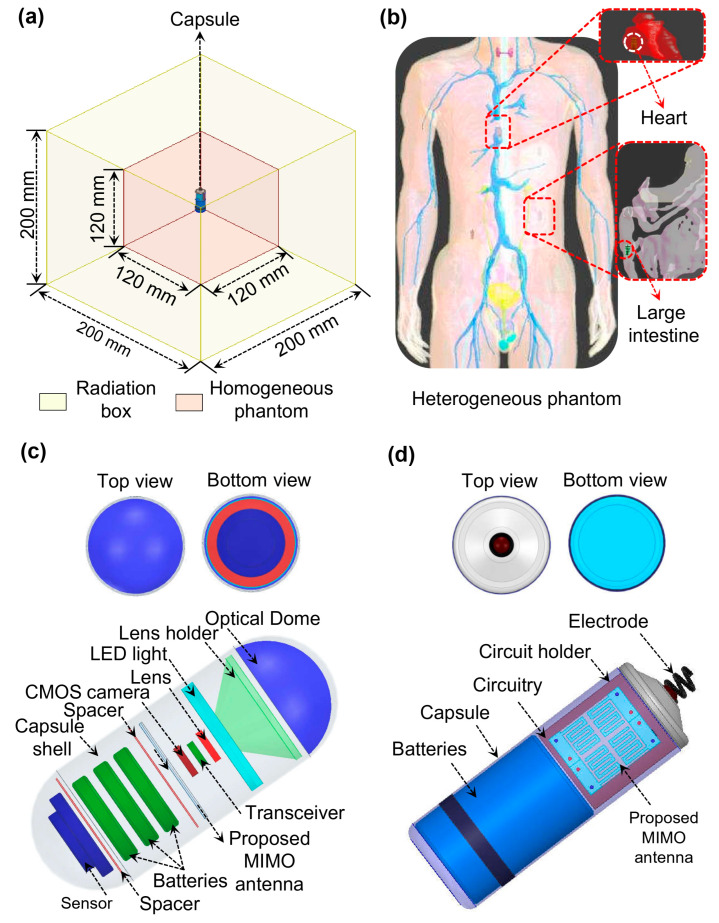
(**a**) Homogeneous simulation setup; (**b**) heterogeneous simulation setup; (**c**) top, bottom, and 3D view of wireless capsule endoscopy (WCE); (**d**) top, bottom, and 3D view of leadless cardiac pacemaker devices.

Biocompatible layers are typically applied to implantable antennas to isolate them from adjacent biological tissues. Nevertheless, beyond material-level considerations, real-time deployment requires the antenna to function as part of a complete implantable system. To address this practical requirement, capsule-type implantable devices were developed in this work, incorporating essential elements such as the power source, sensing modules, and associated electronic circuitry. The antenna was subsequently positioned inside these devices, and all structural modifications and optimization procedures were carried out with these internal components in place. As a result, the evaluated performances, including impedance matching, antenna coupling, radiation characteristics, and MIMO behavior, include the electromagnetic effect arising from the surrounding device components. This design methodology closely mimics actual implantation conditions, where the antenna operates as an integral part of a fully enclosed medical device prior to placement within the human body. The structural configurations of both implants are illustrated in Figure 2. The air-filled WCE capsule has a total length of 24.65 mm and a radius of 5.5 mm, consisting of two lids of 5.5 mm each and a central cylindrical section of 13.65 mm, as shown in Figure 2c. The air-filled leadless pacemaker measures 24.5 mm in length with an 11 mm diameter and incorporates a 1.2 mm upper lid, as shown in Figure 2d. Both devices have 0.25 mm-thick walls designed from aluminum oxide (Al_2_O_3_) with ε_r_ = 9.8 and tanδ = 0.006. Their internal space accommodates essential electronic components such as batteries, sensors electrodes, cameras, and the proposed dual-band MIMO antenna. For simulation purposes, the electronic circuitry is designed by a substrate layer positioned between two perfect electric conductor (PEC) plates, modeled using Rogers RT/duroid 6010 (ε_r_ = 10.2, tanδ = 0.0023, 0.4 mm thick). The antenna is designed and analyzed in HFSS and Sim4Life within a specialized in-body simulation environment. To ensure realistic implantation conditions, it is positioned at the center of a homogeneous large-intestine phantom with frequency-dependent dielectric properties of the tissue. The dielectric properties, including the relative permittivity (ε_r_) and electrical conductivity (σ) of the tissues at both operational frequencies, are summarized in Table 2. The phantom measures 120 × 120 × 120 mm^3^ and is enclosed by a 200 × 200 × 200 mm^3^ radiation boundary, with an implantation depth of 60 mm, as shown in Figure 2a. Validation in heterogeneous biological tissues is further performed in Sim4Life at the same implantation depth, as depicted in Figure 2b.

## 3. Design Evolution and Performance of SISO 

**Figure 3:**
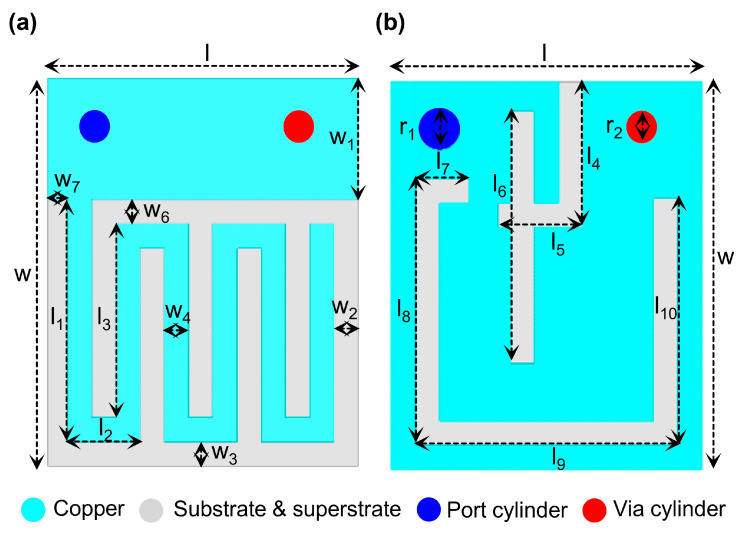
(**a**) Top view of Ant-I (**b**) bottom view of Ant-I.

The MIMO antenna development begins with the design of a single-element SISO antenna (Ant. I), illustrated in Figure 3a,b, with dimensions of 4.0 × 3.2 × 0.5 mm^3^. The structure consists of a radiating patch, shorting pin, superstrate, substrate, and ground plane. Miniaturization is achieved through the use of high-permittivity Rogers RT/duroid 6010 (ε_r_ = 10.2, tanδ = 0.0023) and the partial meandered structure of the patch. An identical superstrate layer is incorporated to reduce coupling with internal device components. A gap of 0.25 mm (w_2_ and w_3_) is maintained intentionally between the patch edge and the adjacent slots on each side of the radiating element to reduce coupling between closely connected elements in the MIMO system. The antenna is excited using a 50 Ω coaxial feed with a 0.4 mm diameter.

**Figure 4:**
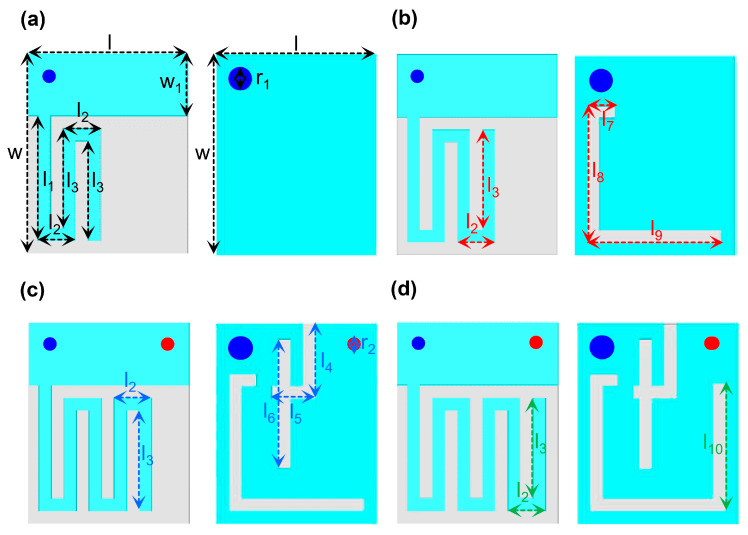
Design evolution of dual-band Ant-I: (**a**) step I, (**b**) step II, (**c**) step III, and (**d**) step IV.

**Figure 5:**
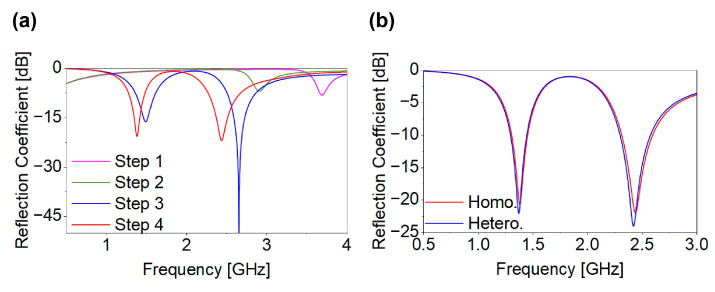
(**a**) Performance evolution of Ant-I; (**b**) |S_11_| of Ant-1.

A four-step parametric optimization procedure was employed to achieve the targeted dual-band performance of Antenna I. Figure 4 illustrates the design evolution of the proposed dual-band Ant-I MIMO element. The initial configuration, as shown Figure 4a, consists of a square radiating patch incorporating three rectangular slots (l_1_–l_3_) above a full ground plane, producing a single resonance at 3.69 GHz with |S_11_| = −8.13 dB. In step II, as shown in Figure 4b, two additional patch slots with dimensions identical to l_2_ and l_3_, along with three ground slots (l_7_–l_9_), are introduced. These modifications increase the effective current path length, thereby shifting the resonance to 2.90 GHz with |S_11_| = −6.98 dB. Step III, as shown in Figure 4c, further incorporates two additional patch slots, a shorting pin (r_2_), and three ground slots (l_4_–l_6_), resulting in the excitation of dual resonance at 1.49 GHz and 2.65 GHz with |S_11_| values of −16.19 dB and −50.18 dB, respectively. Finally, in step IV, as shown Figure 4d, two more patch slots and an additional ground slot (l_10_) are introduced to fine-tune impedance matching, achieving the desired dual-band operation at 1.4 GHz and 2.45 GHz with |S_11_| values of −20.67 dB and −21.9 dB. Throughout the design evolution, the antenna maintains linear polarization. The reflection coefficient performance in both homogeneous and heterogeneous environments, as shown in Figure 5b, shows good agreement. The stepwise design evolution is presented to provide physical insight into resonance formation and impedance tuning mechanisms. The geometrical parameters of the SISO antenna are listed in Table 3.

## 4. Two- and Four-Port MIMO Antenna Design and Analysis

**Figure 6:**
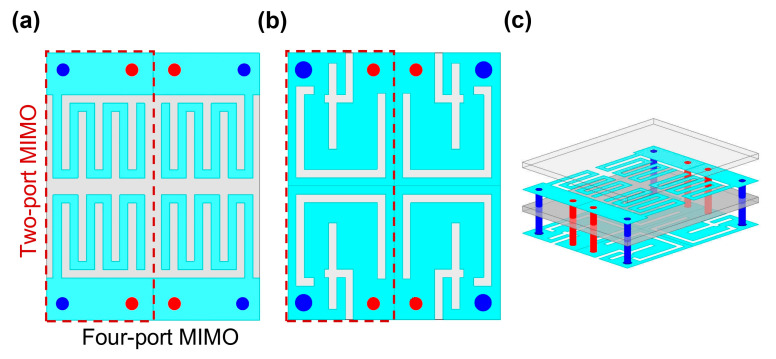
MIMO antenna. (**a**) Top view; (**b**) bottom view; (**c**) exploded 3D view.

**Figure 7:**
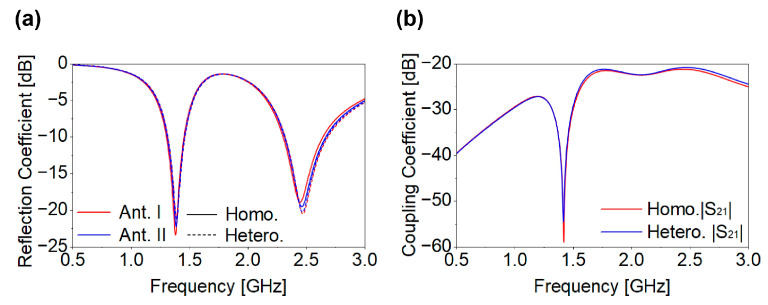
Two-port MIMO antenna: (**a**) reflection coefficient; (**b**) coupling coefficient.

**Figure 8:**
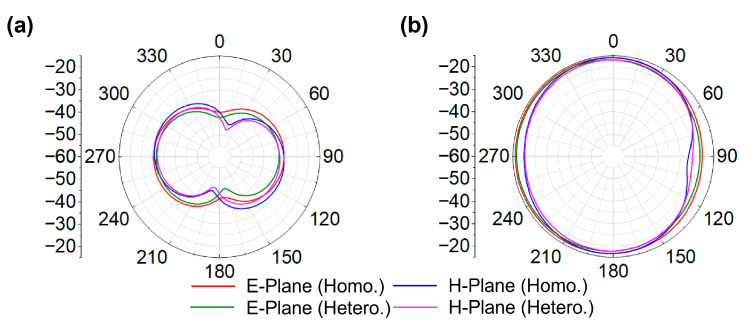
Radiation pattern of two-port MIMO antenna: (**a**) 1.4 GHz; (**b**) 2.45 GHz.

**Figure 9:**
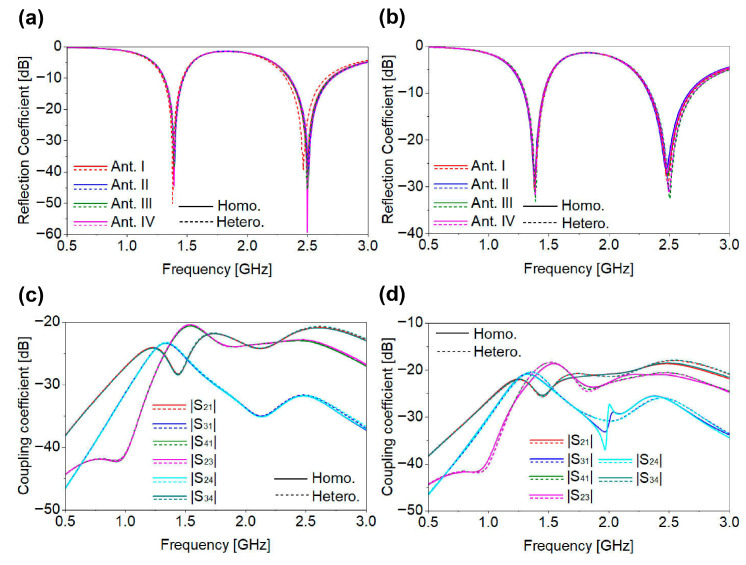
Performance of four-port MIMO antenna: (**a**) reflection coefficient for intestine; (**b**) reflection coefficient for heart; (**c**) coupling coefficient for intestine; (**d**) coupling coefficient for heart.

After a comprehensive parametric study, the optimized Ant-I element was incorporated into the MIMO configuration. Two identical SISO antennas were positioned in opposite orientations, as illustrated in Figure 6. The design of an implantable MIMO system is inherently challenging, as multiple closely spaced elements must be simultaneously tuned while minimizing mutual coupling. As shown in Figure 7a,b, the resulting two-port MIMO antenna achieved the intended resonances without detuning and achieved the desired reflection and coupling coefficients. Mutual coupling remained below −20 dB at both resonant frequencies, as shown in Figure 7b. Figure 8 shows the radiation pattern of a two-port MIMO antenna. At 1.4 GHz, the two-port MIMO antenna mainly radiates between two planes, while at 2.45 GHz it exhibits nearly uniform radiation in all planes, resulting in an omnidirectional-like pattern. The simulated peak gains within biological tissue are −30 dBi and −15 dBi at 1.4 GHz and 2.45 GHz, respectively. The designed two-port MIMO system is encapsulated within the capsule device and evaluated inside the large intestine phantom to validate its in-body operational behavior. Based on the two-port MIMO design and its good reflection and coupling coefficient performance, a four-port MIMO configuration was subsequently developed. The proposed 4 × 4 MIMO antenna is formed by employing the same optimized single-antenna (Ant-I) elements and arranging them in opposite orientation. Accordingly, the reflection coefficients (|S_11_|, |S_22_|, |S_33_|, and |S_44_|) and mutual coupling parameters for all antenna elements demonstrate consistent S-parameter performance. The proposed four-port MIMO system occupies compact dimensions of 8.0 × 6.4 × 0.5 mm^3^, corresponding to a total volume of 25.6 mm^3^. The antenna footprint was selected to ensure compatibility with capsule-type implants, as discussed in Section 2. The proposed structure meets the spatial constraints of both WCE and cardiac leadless pacemaker devices, enabling seamless integration, as illustrated in Figure 2c,d. To validate its applicability for WCE and cardiac pacemakers, the four-port MIMO antenna was evaluated within both large intestine and heart phantoms. Figure 9 presents the reflection and coupling coefficients under homogeneous and heterogeneous tissue conditions. In the intestine phantom, reflection coefficients range from −27.21 dB to −32.15 dB at 1.4 GHz and from −18.45 dB to −29.05 dB at 2.45 GHz, as shown in Figure 9a. In the heart phantom, values vary from −20.89 dB to −26.76 dB at 1.4 GHz and −19.26 dB to −23.12 dB at 2.45 GHz, as shown in Figure 9b. All ports maintain stable resonances and satisfy the required impedance characteristics. High coupling in MIMO antenna systems can significantly degrade overall system performance, particularly in terms of signal isolation, diversity gain, and spatial diversity. This issue is especially critical in biomedical applications, where maintaining reliable transmission quality is challenging due to the lossy nature of human body tissues. Recent studies [29,30] have demonstrated the use of an inductor between two antennas for biomedical applications to achieve a coupling level below −15 dB. Integrating inductors is an effective approach to reduce coupling their implementation presents significant challenges, such as increases in fabrication complexity and integration difficulties. In this study, we achieved the desired isolation without introducing any modifications to the antenna’s structure after MIMO configuration generation and without employing external components. The inter-element spacing in the proposed 4 × 4 MIMO antenna is determined by the predefined geometry of the single-antenna element.

**Figure 10:**
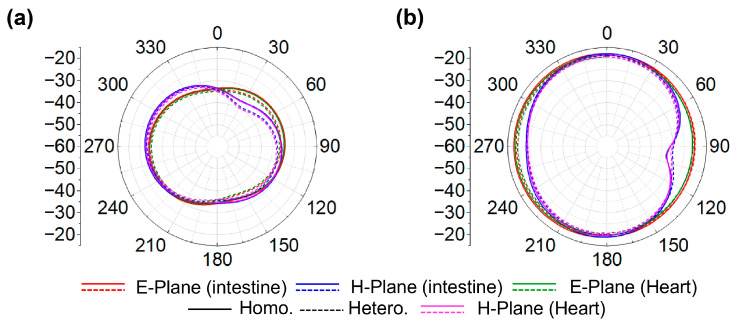
Radiation pattern of four-port MIMO antenna: (**a**) 1.4 GHz; (**b**) 2.45 GHz.

When identical elements are arranged with opposite orientation in the realization of MIMO configuration, the combined edges of adjacent elements yield an effective inter-element spacing of 0.5 mm. This inherent spacing enables efficient suppression of mutual coupling without introducing additional spacing or external decoupling structures. Consequently, compactness is maintained while achieving the desired isolation. In prior works [29,30], using 2 × 2 antenna configurations, inductors were integrated to reduce coupling. In contrast, the proposed 4 × 4 MIMO antenna uses a strategic inter-element spacing of 0.5 mm, achieving the desired isolation without additional components. Despite having more elements, the overall antenna size remains smaller than the referenced 2 × 2 designs [29], due to the optimized geometry and spacing. This approach balances the tradeoff between element spacing, mutual coupling, and compactness, providing a simple, effective and practical solution for implantable devices. In the proposed MIMO antenna, the coupling coefficients |S_21_|, |S_31_|, |S_41_|, |S_23_|, |S_24_|, and |S_34_| remain below −20 dB for both phantoms across the respective frequency bands, as illustrated in Figure 9c,d, well beyond the typical threshold of −15 dB. Due to symmetry (|Smn| = |Snm|), the remaining coefficients are omitted. The far-field radiation characteristics in both simulation environments exhibit good agreement, as shown in Figure 10a,b. At both frequencies, the proposed four-port MIMO antenna exhibits nearly uniform radiation in all planes, resulting in an omnidirectional-like pattern. Since all antenna elements have identical geometry and loading conditions, their radiation characteristics are inherently the same; therefore, the radiation pattern of a single-antenna element is shown as a representative result. The simulated peak gains are −27.5 dBi for the intestine and −26.3 dBi for the heart at 1.4 GHz, and −17.5 dBi for both tissues at 2.45 GHz. The slightly lower gain observed in the intestine is attributed to the substantial electromagnetic attenuation caused by lossy, high-water-content tissues and the complex anatomical structure of the GI tract, which introduces additional propagation challenges. At 1.4 GHz, the gains are lower because biological tissues exhibit higher dielectric losses at lower frequencies.

## 5. SAR Evaluation for Safety Insurance

**Figure 11:**
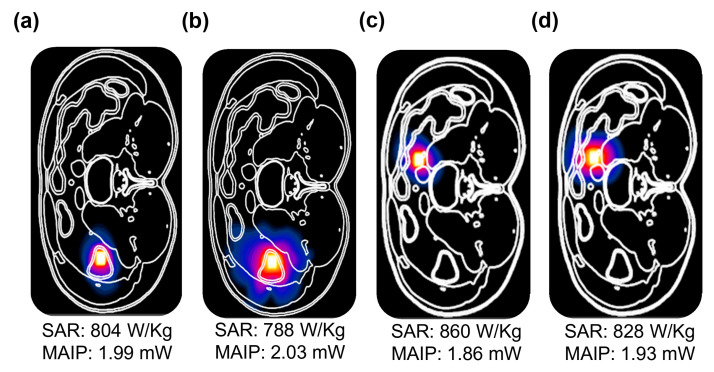
Normalized 1 g SAR distribution and MAIP of the proposed MIMO antenna: (**a**) intestine at 1.4 GHz; (**b**) intestine at 2.45 GHz; (**c**) heart at 1.4 GHz; (**d**) heart at 2.45 GHz.

To ensure patient safety, the electromagnetic exposure of the proposed implantable MIMO antenna was evaluated. The SAR is a key parameter for assessing the biological effects of EM fields on human tissues. According to IEEE C95.1–2019, the maximum permissible SAR is 1.6 W/kg for 1 g tissue and 2 W/kg for 10 g tissue [31]. For the calculation of SAR values, a variety of commercially available simulation tools have been employed in recent years. Notable examples include the heterogeneous human voxel model (Gustav) in CST, the 3D human voxel model in Remcom, and Sim4Life. The SAR analysis was performed using the Sim4Life platform with the anatomically realistic Duke human body model. To reduce computational complexity, the simulation domain was limited to the anatomical region of interest by enclosing the target body section within a bounded computational box and applying controlled padding. A finite-difference time-domain (FDTD)-based solver was employed, with locally refined mesh settings applied to the implantable antenna to ensure accurate geometric representation and precise electromagnetic field resolution. In this study, the SAR was computed with the MIMO antenna placed within heterogeneous heart and large intestine phantoms, as shown in Figure 2b, with each element excited at 1 W input power. Figure 11 presents the resulting SAR values, corresponding maximum allowable input powers (MAIPs), and normalized SAR distributions for both phantoms at their respective resonating frequencies. Given the typical implantable system power requirements (−16 dBm), the antenna operates well within safe limits across both operational frequency bands.

## 6. Data Telemetry Analysis of the Proposed MIMO Antenna

The wireless telemetry performance of the proposed antenna was evaluated through detailed transmission analysis. For the uplink, the implantable antenna operates as the transmitting T_x_ element. The link model is expressed by the following equation:

(1) $$LM=P_{a}-P_{r}$$

where LM denotes the link margin, P_a_ is the available power, and P_r_ is the received power. The available power is computed as follows:

(2) $$P_{a}=P_{T}+G_{T}+G_{R}-L_{p}-PL$$

where PL, the path loss, can be expressed as follows:

(3) $$PL\left(dB\right)=10\gamma {log}_{10}\left(\frac{d}{d_{0}}\right)+20{log}_{10}\left(\frac{4\pi d_{0}}{\lambda_{0}}\right)+X\sigma$$

**Figure 12:**
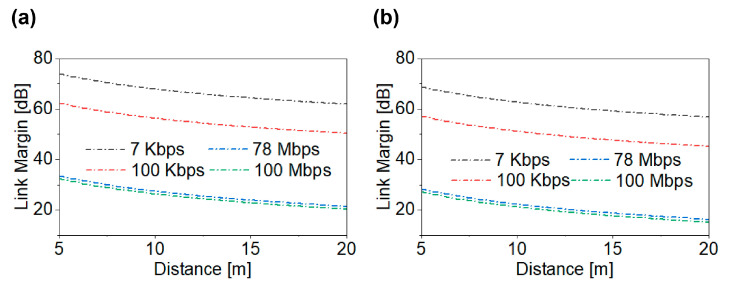
Link margin analysis: (**a**) 1.4 GHz; (**b**) 2.45 GHz.

Here, *γ* denotes the path loss exponent, d_0_ is the reference distance (d_0_ < d), d is the separation between the T_x_ and R_x_ antennas, λ_0_ is the free-space wavelength, and *X*_*σ*_ corresponds to the shadowing component. The link performance analysis is governed by the key communication parameters, including operating frequencies of 1.4 GHz and 2.45 GHz, transmit power *P*_*T*_ = −16 dBm, transmitter antenna gains *G*_T_ of the proposed MIMO antenna, and receiver gain *G*_*R*_ = 2 dBi. The transmission distance was evaluated over a range of 0–20 m. The receiver noise power was calculated using the thermal noise formulation, where *k* = 1.38 × 10^−23^ is Boltzmann’s constant, *T* = 273 K is the reference temperature, and *B* represents the communication bandwidth corresponding to each data rate. The modulation implementation margin for quadrature phase shift keying (QPSK) was considered as 9.6 dB. As illustrated in Figure 12a,b, for data rates of 7 Kbps, 100 Kbps, 78 Mbps, and 100 Mbps, the proposed MIMO antenna supports a reliable high-speed (100 Mbps) link exceeding 20 m at both operating frequencies. The antenna gain used in link analysis corresponds to the realized gain obtained from electromagnetic simulation, while the path loss model represents large-scale propagation attenuation and does not separately model microscopic tissue absorption to avoid double counting of loss mechanisms.

## 7. MIMO Channel Parameters

The performance evaluation of an MIMO antenna system, comprising multiple radiating elements, requires a more comprehensive analysis than that of SISO designs. In particular, inter-element correlation must be analyzed to determine the system’s practical suitability. Key parameters, including the ECC, DG, CCL, and TARC, are critical for evaluating the performance of the antenna’s elements within the MIMO configuration.

### 7.1. ECC and DG

**Figure 13:**
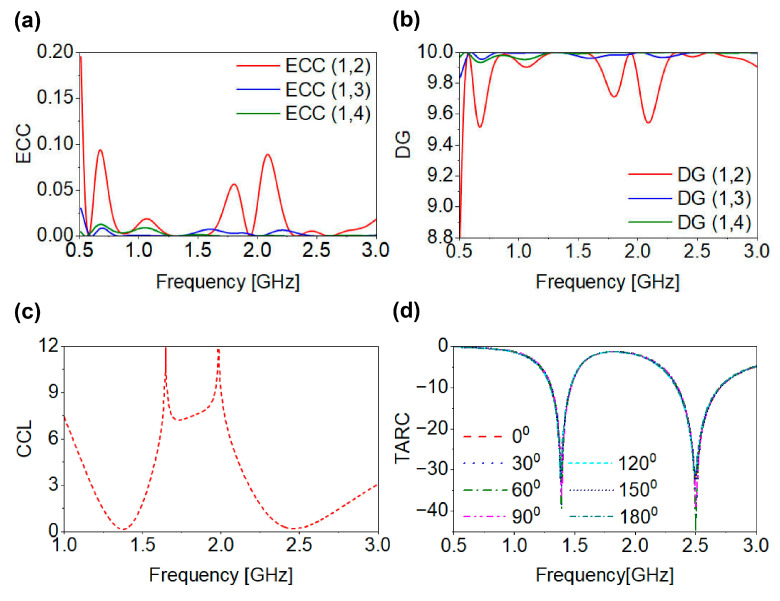
MIMO channel parameters: (**a**) ECC; (**b**) DG; (**c**) CCL; (**d**) TARC.

The ECC calculates the correlation between closely spaced antenna elements in an MIMO system. Although zero correlation is theoretically desired, this condition is not practically attainable; therefore, ECC values below 0.5 are generally considered acceptable. The ECC can be calculated from either S-parameters or radiation patterns. While S-parameter-based calculations are widely used, they are valid only for lossless antennas in isotropic environments. Printed antennas, particularly those operating inside the human body, exhibit significant losses, which can lead to inaccurate ECC calculation when relying on S-parameters. In contrast, the radiation-pattern-based method provides a more accurate representation because it reflects the true channel characteristics, considering antenna losses, spatial behavior, and mutual coupling effects. It also maintains the actual radiative properties and their influence on channel performance, making it a more reliable evaluation method. In this study, the ECC is determined using the far-field radiation patterns of the proposed antenna using the following equation:

(4) $$ECC=\frac{{\left|\iint_{0}^{4\phi }\left[{\vec{A}}_{xi}\left(\theta ,\;\phi \right)\times \;{\vec{A}}_{yj}\left(\theta ,\;\phi \right),\;d\Omega \right]\right|}^{2}}{\iint_{0}^{4\phi }{\left|{\vec{A}}_{xi}\left(\theta ,\;\phi \right)\right|}^{2},\;d\Omega \;\iint_{0}^{4\phi }{\left|{\vec{A}}_{yj}\left(\theta ,\;\phi \right)\right|}^{2},\;d\Omega }$$

where ${\vec{A}}_{xi}$ and ${\vec{A}}_{yj}$ are the 3D radiation patterns. As shown in Figure 13a, the ECC values are lower than the standard 0.5 value across all bands. These low ECC values indicate excellent isolation within the proposed MIMO system, confirming its suitability for implantable medical devices that require reliable, high-data-rate communication. The DG is another significant parameter that calculates the improvements in the signal-to-interference ratio achieved through diversity techniques, ensuring rigorous antenna performance without losses. A DG value of 10 dB is generally considered optimal, corresponding to correlation between antenna elements. The DG can be computed using the following equation:

(5) $$\mathrm{DG}=10\sqrt{1-{ECC}^{2}}$$

Figure 13b presents the DG values of the proposed MIMO antenna. The DG values approaching 9.9 dB across both frequency bands indicate excellent diversity performance, confirming the antenna’s suitability for applications requiring reliable and robust communication.

### 7.2. CCL and TARC

The CCL represents a critical parameter in MIMO systems, defining the ultimate limit on the data transmission rate achievable across the communication channel. It is calculated as follows:

(6) $$CCL=-{log}_{2}det(\alpha^{R})$$

where $\alpha^{R}$ denotes the correlation matrix, defined as follows:

(7) $$\alpha^{R}=\;\left[\begin{array}{cccc} R_{11} & R_{12} & R_{13} & R_{14}\\ R_{21} & R_{22} & R_{23} & R_{24}\\ R_{31} & R_{32} & R_{33} & R_{34}\\ R_{41} & R_{42} & R_{43} & R_{44} \end{array}\right]$$

The diagonal $R_{ii}$ and off-diagonal $R_{ji}$ elements of the correlation matrix $\alpha^{R}$ are provided as follows:

(8) $$R_{ii}=1-\sum_{j=1}^{N}|S_{ij}{|}^{2}\;and\;R_{ij}=\;-\left(S_{ii}^{*}S_{ii\;}+\;S_{ji}^{*}S_{jj\;}\right)\;for\;i,\;j=\;1,\;2,\;3,\;4$$

Figure 13c shows the CCL performance, with values of 0.21 b/s/Hz and 0.18 b/s/Hz at the lower and higher resonant bands. Both remain well below the 0.4 b/s/Hz threshold, confirming the good channel performance of the proposed MIMO system. The ECC was evaluated using the radiation pattern method to provide an accurate estimation of spatial diversity performance in lossy biological environments. In contrast, CCL was computed using the S-parameter-based correlation matrix formulation, which is widely adopted for evaluating coupling-induced channel capacity degradation in compact MIMO antenna systems. This distinction ensures that the ECC and CCL quantify different performance aspects, where the ECC represents spatial radiation correlation while CCL reflects port coupling and network-level capacity reduction. The TARC is another important parameter for evaluating MIMO performance, as it calculates the ratio of total reflected power to total incident power across all antenna elements during simultaneous multi-port operation. For the proposed four-port MIMO antenna, the TARC is defined as follows:

(9) $$TARC=\frac{\sqrt{\sum_{n=1}^{N}|S_{i1}+\sum_{m=2}^{N}S_{\mathrm{im}e^{j(\theta_{m}-1)}}{|}^{2}}}{\sqrt{N}}$$

Figure 13d presents the TARC values for phase excitations of 0–180°. In all cases, the TARC remains below −10 dB at both resonant bands, confirming stable multi-port operation. The proposed four-port MIMO configuration is developed using the initially designed and optimized single-antenna element. While a single element can support data transmission within the allocated bandwidth, the planar four-port MIMO configuration provides significant advantages in terms of spatial diversity and channel robustness, particularly in lossy implantable environments. The achieved low ECC, high DG, and low CCL indicate improved signal reliability and effective channel utilization compared to a single-port configuration. These characteristics enhance communication stability and support higher reliable data throughput. Overall, the MIMO parameter analysis demonstrates good system performance, making the proposed antenna a promising candidate for high-data-rate biomedical applications such as capsule endoscopy and leadless pacemakers.

## 8. Conclusions

This work presented a highly miniaturized four-port dual-band implantable MIMO antenna for capsule endoscopy and cardiac leadless pacemakers operating in the WMTS and ISM bands. Through a compact meandered structure, the antenna achieves an overall volume of 25.6 mm^3^ while maintaining good performance inside realistic large intestine and heart phantoms. The design demonstrates good reflection coefficients of −31.19 dB and −30.07 dB, gains of −27.5 dBi and −17.5 dBi, −10 dB impedance bandwidths of 170 MHz and 370 MHz, mutual coupling below 20 dB, and fractional bandwidths of 12.2% and 15.1% at 1.4 GHz and 2.45 GHz, respectively. Extensive full-wave electromagnetic simulations were performed using homogeneous and heterogeneous body phantoms, including fully encapsulated implants. The study covered the design and optimization of a single-antenna element and its extension to two- and four-element MIMO configurations, and analysis including homogeneous and heterogeneous reflection coefficients, coupling coefficients, and omnidirectional-like radiation patterns. SAR evaluation confirms compliance with safety limits, and wireless telemetry analysis verifies reliable high-data-rate link performance. Furthermore, MIMO channel parameters, including the ECC, DG, CCL, and TARC, validate the antenna’s capability for multi-port operation. Overall, the proposed MIMO antenna offers a miniaturized, safe, and efficient solution for high-data-rate implantable biomedical devices. Experimental validation of S-parameters, radiation patterns, and received power will be addressed in future work. 

## Figures and Tables

**Table 1:** Comparison with reported MIMO implantable antennas.

References	Size [mm^3^]	No. of Elements	Frequency [GHz]	Isolation [dB]	Gain [dBi]	Depth [mm]	Antenna Profile	Tissue
[12]	307	4	0.403	−26	−36	4	Planar	1-layer
[13]	441.27	4	2.45	−15.9	−15.2	19.5	Planar	3-layer
[14]	120.58	4	1.4 2.45	−23 −24	−28.17 −18.15	50	Cubic	Heart, GI
[15]	280.035	4	0.433	−37	−21.8	3	Planar	1-layer
[16]	3375	4	2.45 5.8	−37 −32	−18.5	50	Cubic	3-layer
[17]	301.716	4	1.47 2.45	>−18.15 >−21.1	−27.09 −29.73	25	Planar	GI
[24]	28.1	2	0.915, 2.45	35.85, 31.6	−29.8 −24.6	50	Planar	GI
[25]	14.7	2	0.915, 2.45	>35 >27	−30.47 −24.71	17	Planar	Brain
This work	25.6	4	1.4 2.45	−27.7 −31.8	−27.5 −17.5	60	Planar	Heart, GI

Size (mm^3^) represents the physical volume of the antenna’s structure. GI tissue refers to gastrointestinal tissue.

**Table 2:** Human body tissue properties at resonant frequencies.

Frequency	1.4 GHz		2.45 GHz	
Tissue	$\varepsilon_{r}$	σ	$\varepsilon_{r}$	σ
Heart	57.5	1.51	54.8	2.26
Large intestine	56.1	1.33	53.9	2.03

**Table 3:** Geometrical parameters of single-antenna element.

Variables	Values [mm]	Variables	Values [mm]	Variables	Values [mm]
l	4	l_1_	2.5	l_3_	2
w	3.2	l_2_	0.5	w_1_	1.25
w_1_–w_5_	0.25	w_6_	0.2	l_6_	2.6
l_7_	0.55	l_8_	2.75	l_9_	2.7
l_10_	2.55	r_1_	0.2	r_2_	0.15

## Data Availability

The data are available upon reasonable request.

## Abbreviations

SISO	Single-Input Single-Output
MIMO	Multiple-Input Multiple-Output
WCE	Wireless Capsule Endoscopy
WMTS	Wireless Medical Telemetry Service
ISM	Industrial, Scientific and Medical
SAR	Specific Absorption Rate
MAIP	Maximum Allowable Input Power
ECC	Envelope Correlation Coefficient
DG	Diversity Gain
CCL	Channel Capacity Loss
TARC	Total Active Reflection Coefficient
